# Ambulance service satisfaction level and associated factors among service users in Addis Ababa, Ethiopia

**DOI:** 10.1186/s12873-024-01007-9

**Published:** 2024-05-31

**Authors:** Fisseha Zeleke Asfaw, Ayalnesh Zemene Yalew, Mezgebu Godie, Ayele Fikadu, Abdata Workina

**Affiliations:** 1 Department of Emergency and Critical Care, Saint Paul Hospital Millennium Medical College, Addis Ababa, Ethiopia; 2 School of Nursing, Saint Paul Hospital Millennium Medical College, Addis Ababa, Ethiopia; 3 School of Nursing, Jimma University, Oromia, Ethiopia

**Keywords:** User satisfaction, Ambulance service, Emergency medical services, Pre-hospital

## Abstract

**Introduction:**

Pre-hospital ambulance service is the most important part of healthcare service. Client satisfaction with the service indicates the degree of adaptation to the appropriate quality and quantity of services. Patients’ dissatisfaction with the service can affect their expectations of the overall services that they will receive later in the definitive care facility. However, it is not a well-addressed area in developing countries, including Ethiopia.

**Objective:**

This study aimed to identify the ambulance service satisfaction level and associated factors among service users in Addis Ababa, Ethiopia.

**Methods:**

A cross-sectional study was conducted in five governmental hospitals in Addis Ababa city. A face-to-face exit interview technique was employed on a total of 410 consecutively selected participants using a pretested tool developed from similar sources. The cleaned data was entered into the Epi-Data Manager 4.6 version and then exported to SPSS version 26 for analysis. The dependent variable was dichotomized into satisfied and unsatisfied to compute bivariate logistic regression. In the multivariate logistic regression model, predictors with a *p*-value < 0.05 at the 95% CI were considered to have a significant association.

**Result:**

A total of 410 respondents were included in the study. The mean of participants’ responses regarding ambulance personnel, call operator, treatment on the scene, and ambulance subscale was 3.64, 3.48, 3.40, and 3.43, respectively. The study found that only 21.5% of participants were satisfied by the ambulance service they received. There was a statistically significant association between ambulance service satisfaction and age (AOR = 3.52, 95% CI: 1.01–12.36), monthly income (AOR = 3.13, 95% CI: 1.41–6.94), ambulance response time (AOR = 10.33, 95% CI: 2.09–51.06), type of ambulance used (AOR = 4.55, 95% CI: 2.19–9.43), and previous ambulance usage (AOR = 2.33, 95% CI: 1.34–4.05).

**Conclusion:**

The study found a low level of satisfaction among ambulance users. The findings suggest that ambulance personnel performance is a key determinant of user satisfaction, while treatment at the scene and in the ambulances, and call operator areas require improvement. Age, monthly income, ambulance response time, type of ambulance, and previous ambulance use also influenced satisfaction. Improving the quality of services, reducing response time, and ensuring call operators are trained are vital steps to enhance satisfaction.

## Introduction

Emergency Medical Service, also called ambulance service, paramedic service, or pre-hospital service, is one of the ways of addressing the proper scene and en-route assessment with the appropriate corresponding management when an emergency occurs [1–3]. Apart from providing emergency healthcare services, it also plays a great role in the safe and quick transportation of victims from the scene to a definitive care facility or from facility to facility [2, 4, 5].

Patient satisfaction is a component of healthcare services that has a distinct value to the patient and conceptualizes the difference between how the patient expected to be attended to and how the patient was attended [6, 7]. Assessing patients’ satisfaction with the service provision is mandatory since patient satisfaction is seen as a healthcare outcome and can forecast adherence to treatment, care, and support [8, 9]. As patient satisfaction is client-oriented in nature, it also provides insight into patients’ subjective experiences of the service that cannot be detected otherwise [4].

Globally, among the vast variety of components of the healthcare system, urgency and emergency health services were given priority in health institutions’ qualification processes, and these services were recognized as one of the main sources of complaints by the population [6]. EMSs (emergency medical services) are the most important part of the healthcare system; patient satisfaction indicates the degree of adaptation of appropriate quality and quantity of services subjectively from their perspective [10–12]. Besides, identifying satisfaction and dissatisfaction with emergency care services might be represent patients’ views about the overall condition of the healthcare delivery system [11, 13]. Ambulance users’ satisfaction is a measure of the quality of healthcare service, and its findings might be utilized to evaluate the structure, procedures, and outcomes attained regarding services [6, 14].

Even though some studies were conducted regarding prehospital ambulance services in developed countries, there has been no scientific study conducted in Ethiopia on ambulance service users’ satisfaction. However, some related literatures studied the satisfaction of patients regarding in-hospital services such as maternity care, emergency department, and patient referral systems [1, 15–19]. The model of emergency care service in Ethiopia is a Franco-German model in which almost all patients are transported to the emergency department, and the care is rendered by an emergency medical technician and trained nurses on prehospital care in some parts of the country. A study conducted on users’ satisfaction with maternity waiting home services in Ethiopia elucidated that users’ overall satisfaction with the service was moderate; besides, most services and standards of the service were lower extremes of satisfaction dimensions [17]. Thus, the study’s research questions were: (1) what is the level of prehospital emergency care ambulance service satisfaction among consumers? (2) What are the factors that affect ambulance service satisfaction among service users? Thus, this study aimed to identify patient satisfaction and associated factors among service users.

## Patients and methods

### Study design and setting

A cross-sectional study design was employed among randomly selected five governmental hospitals (Addis Ababa Burn Emergency and Trauma Hospital, Saint Peter Hospital, Saint Paul Hospital, Black Lion Hospital, and Alert Hospital) in Addis Ababa from May 1 to July 12, 2023. Addis Ababa is the capital and largest city of Ethiopia [20]. As of 2022, the city has 53 hospitals, of which 13 are government-owned and 40 are private. These governmental hospitals have a great deal of emergency patient and ambulance flow to their emergency departments. They cumulatively provide emergency care services for around 3000–4500 patients and have around 600–900 ambulance visits per month at their emergency departments.

### Study populations

The study population consisted of all clients and their attendants who came by ambulance to the selected public hospitals during the study period.

### Eligibility criteria

All clients and patients who traveled to the randomly selected hospitals via ambulance service were included in the study. For unconscious, critical, and < 18-year-old patients, their immediate person or attendant who traveled with them via ambulance to the study settings were considered respondents to the questionnaire. The study excluded patients who transferred out before their illness stabilized. Comatose and dead-on-arrival patients who arrived at the selected hospitals alone or with a medical professional as an attendant were also excluded from the study.

### Sample size and sampling procedure

The sample was calculated using a single population proportion formula by considering the sample size for the proposed study, which was calculated by taking a 5% margin of error (d) and a 95% confidence interval, 50% (P) proportions of ambulance users’ satisfaction since there is no similar study published in the country, and a 10% non-response rate. The sample size for the study was determined as follows:

$${\rm{n}}\left({{\rm{sample}}\,{\rm{size}}} \right){\rm{ = }}\frac{{(Za/2)2\,P(1 - p)}}{{d2}}\, = 423$$

Five government hospitals were selected by using a simple random sampling technique from 13 government hospitals. Then, samples for each hospital were proportionally allocated by considering the total number of ambulance arrivals per month as the source population. The approximate number of ambulance arrivals per month to Addis Ababa Burn Emergency and Trauma Hospital, Saint Peter Hospital, Saint Paul Hospital, Black Lion Hospital, and Alert Hospital were 70, 200, 100, 230, and 120, respectively. The proportional allocated sample size for each hospital was 41, 117, 60, 135, and 70 for Addis Ababa Burn Emergency and Trauma Hospital, Saint Peter Hospital, Saint Paul Hospital, Black Lion Hospital, and Alert Hospital by calculating the approximate total of ambulance arrivals per month (720) for selected hospitals. Each study participant was selected by using a consecutive sampling technique until the target sample size was reached at each hospital. The patient or attendant of the patient was interviewed in the study settings at the respondents’ convenience after the clients became clinically stabilized and upon discharge from the hospitals.

### Data collection tool and data collection procedure

An interviewer-based questionnaire’ from a previous study was adopted to collect the data by using the emergency services patient satisfaction scale [21]. It consists of 26 items rated on a 5-point Likert scale, ranging from strongly disagree = 1 to strongly agree = 5, with a maximum total score of 130 (strongly agree to all parameters) and a minimum total score of 26 (strongly disagree to all parameters of emergency medical service satisfaction). The scale contains four sub-scales: ambulance personnel, call operator, treatment on the scene, and technical equipment of the ambulance. This tool was translated into Amharic and Afan Oromo to match the local contexts, and then used as a data collection tool. In this study, the tool also went through a scale reliability test and yielded a Cronbach’s alpha reliability coefficient of 0.94. Data was collected by five BSc nurses after the provision of training on the data collection tool. Data collection took place in the study settings at the respondents’ convenience after clients became clinically stabilized and upon discharge from the hospitals.

### Data quality assurance

To ensure the quality of the data, before the actual data collection, the adopted and translated data collection instrument passed through a test and re-test procedure in a similar contextual setting. Additionally, data collectors received proper training and orientation on the contents of the data collection tool. The data collection tool was also translated into local contextual language to increase the quality of the data collected.

### Data processing and analysis

The cleaned data was analyzed using SPSS 26 versions. Descriptive statistics were computed to describe the characteristics of the participants and the categories of their responses. A dependent variable was dichotomized into satisfied and unsatisfied to compute bivariate logistic regression. Satisfied is defined by a participant with a total score of *≥* 75% on the tool as being satisfied with the ambulance service [1, 13, 19]. Whereas unsatisfied is a participant with a total score of < 75% on the tool is considered unsatisfied with the service [1, 13, 19]. The model was checked using the Hosmer and Lemeshow model goodness-of-fit for its goodness. In the bivariate logistic regression, variables with a *p*-value < 0.25 were transferred into the multivariate logistic regression. In the multivariate logistic regression model, variables with a *p*-value < 0.05 were considered to have a statistically significant association at the 95% confidence level.

### Ethical considerations

The study was conducted according to ethically accepted standards for using humans as research participants. Primarily, a grant of ethical approval and clearance was obtained from the Institutional Review Board of Saint Paul Hospital Millennium Medical College and given to study site hospitals. Furthermore, an ethical issue was addressed by offering information to study participants on their rights to confidentiality, anonymity, voluntary participation, refusal or withdrawal from the study, and obtaining written informed consent from each participant. This study was conducted in accordance with the Declaration of Helsinki.

## Result

### Socio-demographic characteristics of study participants

A total of 410 respondents participated in the study, yielding a 96.9% response rate. Of the study participants, 275 (67.1%) were attendants of the client. Two hundred sixty-two (63.9%) of the study participants were males, and the mean (SD) age of respondents was 35 (10.3), with a higher portion (42.2%) in the age group between 28 and 37 years. Most of the respondents, 166 (40.5%), have a college diploma or higher educational level. (Table 1)

**Table 1:** Socio-demographic characteristics of ambulance service users in Addis Ababa, 2023

Variables		Frequency	Percent
Respondent	Patient	135	32.9%
	Attendant	275	67.1%
Gender	Male	262	63.9%
	Female	148	36.1%
Age group	18–27 years	88	21.5%
	28–37 years	173	42.2%
	38–47 years	105	25.6%
	48 years and above	44	10.7%
Educational status	No formal education	48	11.7%
	Primary	95	23.2%
	Secondary	101	24.6%
	College and above	166	40.5%
Monthly income	< 88 USD	200	48.8%
	88–175 USD	103	25.1%
	> 175 USD	107	26.1%
Marital status	Married	234	57.1%
	Unmarried	176	42.9%

### Ambulance service-related characteristics of respondents’

Around 171 (41.7%) respondents stated that the ambulance response time was 20–39 min, and the majority of study participants, 303 (73.9%), have used governmental ambulance service. The types of illnesses for ambulance utilization among participants were trauma, which accounts for 43.4%, while the remaining were non-traumatic medical and surgical cases. Two hundred seventy-six (67.3%) study participants have never used an ambulance service previously. (Table 2)

**Table 2:** Ambulance service-related characteristics of respondents in Addis Ababa, Ethiopia, 2023

Variables		Frequency	Percent
Ambulance response time	< 9 min	19	4.6
	10–19 min	143	34.9
	20–39 min	171	41.7
	> 39 min	77	18.8
Type of ambulance used	Government ambulance	303	73.9
	Private ambulance	59	14.4
	Non-governmental ambulance	48	11.7
Type of illness for ambulance users	Trauma	178	43.4
	Non-trauma	232	56.6
Previous ambulance use experience	Yes	134	32.7
	No	276	67.3

### Ambulance service satisfaction level

The mean of the participants’ emergency medical services satisfaction scores was 91.73 with an SD of 16.84, with higher satisfaction in the aspects of ambulance personnel and lower satisfaction related to treatment on the scene, technical equipment of the ambulance, and call operators’ subscales. Using 75% as a cut-off point, participants who were satisfied with the ambulance service provided were determined to be 21.5%, while the rest, 78.5%, were unsatisfied with the service. (Table 3)

**Table 3:** Mean, standard deviations and overall ambulance satisfaction level of participant’s response to the 112 Emergency Services Patient Satisfaction Scale in Addis Ababa, 2023

Items	Mean	SD
**Ambulance Personnel**		
The ambulance personnel asked questions about the patient’s/injured person’s complaints.	3.58	1.030
The ambulance personnel listened to the patient’s/injured person’s complaints.	3.27	1.067
The ambulance personnel provided explanatory information about the patient/injured person.	3.08	1.146
The ambulance personnel sufficiently took care of the patient/injured person.	3.80	1.085
The ambulance personnel gave moral support to the patient/patient’s relative.	3.84	1.047
I had confidence in the ambulance personnel’s professional knowledge.	3.66	1.067
I was satisfied with the ambulance personnel’s overall attitude.	3.61	1.059
The ambulance personnel were wearing uniforms.	3.55	1.178
The ambulance personnel respected the hygiene rules.	3.63	0.947
I appreciated the ambulance personnel’s teamwork.	3.69	1.023
The ambulance personnel did their best for us.	3.94	0.908
The ambulance personnel transported the patient/injured person to the hospital as quickly as possible.	4.05	0.979
**Subscale mean**	**3.64**	**1.045**
**Call operator**		
The call operator understood my statements.	3.46	0.961
The call operator was respectful to me.	3.49	0.965
I had confidence in the call operator.	3.50	0.954
**Subscale mean**	**3.48**	**0.960**
**Treatment on the Scene**		
The ambulance personnel gave the needed treatment to the patient/injured person on the scene.	3.53	1.030
The ambulance personnel brought all the devices they would use to the scene.	3.02	1.159
The ambulance personnel safeguarded the patient’s/injured person’s privacy.	3.72	0.926
The ambulance personnel served with a smile.	3.56	1.116
The ambulance personnel gave clear answers to our questions.	3.40	1.068
The ambulance personnel explained the needed procedures.	3.21	1.079
The devices that the ambulance personnel used functioned properly.	3.41	1.062
**Subscale mean**	**3.40**	**1.063**
**Technical equipment of the ambulance**		
The ambulance was equipped enough for all kinds of treatment.	**2.54**	1.072
Inside the ambulance was convenient for weather conditions.	3.50	1.050
Inside the ambulance was quiet, noise-free, and comfortable.	3.58	1.095
Transportation to the hospital by ambulance was provided safely.	4.11	0.871
**Subscale mean**	**3.43**	**1.022**
Satisfaction regarding ambulance services	**Satisfied**	88(21.5%)
	**Unsatisfied**	322(78.5%)

### Factors associated with ambulance service satisfaction

During a binary logistic regression analysis using a 95% CI, eight independent variables were found to be candidates for the final model (*P* < 0.25). These variables were respondent characteristics (being a patient or attendant as a respondent), age, educational level, monthly income, ambulance response time, type of ambulance used, type of illness for ambulance use, and previous ambulance usage. After performing the model goodness-of-fit test, the candidate variables were analyzed through a multivariate logistic regression analysis using a 95% CI. Accordingly, age, monthly income, ambulance response time, type of ambulance used, and previous ambulance usage were statistically significant factors in ambulance service satisfaction. (Table 4)

**Table 4:** Bivariate and multivariate logistic regression of ambulance service satisfaction level among ambulance users in Addis Ababa, 2023

Variable	Category	*n* = 410 (%)	COR (95% CI)	AOR (95% CI)	*p*-value
Respondent	Patient	135 (32.9)	1	1	
	Attendant	275 (67.1)	1.889 (1.092-3,268)	1.185 (0.603 − 2.230)	0.623
Gender	Male	262 (63.9)	1	1	
	Female	148 (36.1)	1.221 (0.752-1.983)	1.068 (0.586-1.948)	0.829
Age	18–27 years	88 (21.5)	3.287 (0.817-13.221)	**3.520 (1.002–12.361)**	**0.050** ^*^
	28–37 years	173 (42.2)	1.872 (0.517-6.776)	1.705 (0.524 − 5.550)	0.376
	38–47 years	105 (25.6)	2.212 (0.640-7.643)	2.283 (0.698-7.467)	0.172
	> 47 years	44 (10.7)	1	1	
Educational status	None	48 (11.7)	0.572 (0.248-1.318)	0.947 (0.310-2.893)	0.923
	Primary	95 (23.2)	0.623 (0.332 − 1.170)	1.165 (0.494-2.743)	0.727
	Secondary	101 (24.6)	0.706 (0.388-1.287)	1.206 (0.530-2.743)	0.655
	**>** College	166 (40.5)	1	1	
Marital status	Married	234 (57.1)	1	1	
	Unmarried	176 (42.9)	0.955 (0.593-1.539)	1.195 (0.628-2.139)	0.637
Monthly income	*≤* 4999	200 (48.8)	1.182 (0.487-2.873)	1.176 (0.536-2.583)	0.686
	5000–9999	103 (25.1)	3.065 (1.348–6.967)	**3.129 (1.412–6.936)**	**0.005** ^*^
	*≥* 10,000	107 (26.1)	1	1	
Ambulance response time	*≤* 9 min	19 (4.6)	10.345(2.067–51.782)	**10.331(2.090-51.064)**	**0.004** ^*^
	10–19 min	143 (34.9)	4.815 (1.630-14.224)	**4.811 (1.671–13.850)**	**0.004** ^*^
	20–39 min	171 (41.7)	5.568 (1.869–16.589)	**5.780 (2.012–16.604)**	**0.001** ^*^
	*≥* 40 min	77 (18.8)	1	1	
Type of ambulance	Governmental	303 (73.9)	1	1	
	NGO	48 (11.7)	4.580 (2.152–9.750)	**4.547 (2.193–9.430)**	**0.001** ^*^
	Private	59 (14.4)	3.380 (1.463–7.811)	**3.681 (1.701–7.964)**	**0.001** ^*^
Type of illness	Trauma	178 (43.4)	1	1	
	Non-trauma	232 (56.6)	2.132 (1.284–3.542)	1.596 (0.856-3.011)	0.148
Previous ambulance use	Yes	134 (32.7)	2.269 (1.223–4.208)	**2.329 (1.338–4.052)**	**0.003** ^*^
	No	276 (67.3)	1	1	

Age groups 18–27 years are 3.5 (AOR = 3.520, 95% CI: 1.002–12.361) times more likely to be satisfied with the ambulance service compared to the age group > 47 years. Individuals with a monthly income between 88 and 175 USD are more than triple (AOR = 3.129, 95% CI: 1.412–6.736) times more likely to be satisfied than those who earned *> 175 USD*. Service utilizers with an ambulance response time of < 9 min (AOR = 10.331, 95% CI: 2.090–51.064), 10–19 min (AOR = 4.811, 95% CI: 1.671–13.850), and 20–39 min (AOR = 5.780, 95% CI: 2.012–16.604) are 10.3, 4.8, and 5.7 times more likely to be satisfied than users with a response time *≥* 40 min, respectively. Private ambulance service users are more than three and a half times (AOR = 3.681, 95% CI: 1.701–7.964) and NGO ambulance users are more than four and a half times (AOR = 4.547, 95% CI: 2.193–9.430) times more satisfied by the service than governmental ambulance service users, and participants who used ambulance service previously are also more than double times (AOR = 2.329, 95% CI: 1.338–4.052) more satisfied than their counterparts. (Table 4)

## Discussion

This study reports client satisfaction and associated factors regarding ambulance service usage in Addis Ababa. In this study, only 21.5% of participants were satisfied with the ambulance service, while the majority (78.5%) were unsatisfied with the service they received. This study’s findings supplement the limitations of prehospital emergency care in Africa that contribute to emergency center mortality. This result of users’ satisfaction considerably contradicts the majority of studies conducted regarding ambulance service satisfaction that produced a ceiling effect stating a high ambulance service user satisfaction portfolio [3, 4, 8, 10, 11, 22–26]. This discrepancy in satisfaction level might be due to the difference in ambulance service quality that exists among developed and developing countries, given that almost all the literature referenced was conducted in well-developed countries and there is a literature gap regarding ambulance service satisfaction in developing countries to compare to. Furthermore, this study finding has the implication that there is insufficient prehospital emergency care that embraces limited treatment on the scene, prolonged response time, and inadequate patient centred emergency medical service care in Ethiopia.

According to the findings of this study, higher satisfaction was seen in aspects of ambulance personnel, and low satisfaction was related to treatment on the scene, technical equipment of the ambulance, and the call operator’s subscales. This finding is in line with Bogomolova [4] and Farhadloo [11] who reported in their study that the highest level of ambulance users’ satisfaction was seen in the area of paramedics’ performance and the lowest rate of satisfaction was seen in the aspect of the ambulance. But opposed to this, Porsuk [3] reported in their study that higher levels of client satisfaction were seen in the area of call operators and the ambulance.

In this study, age showed an association with ambulance service satisfaction. Study participants within the age group of 18–27 years were more satisfied with the service provided than those who were > 47 years old. This result supports the findings of previous studies [3, 4, 27] that also identified a positive relationship between age and ambulance service satisfaction. However, on the contrary, this finding contradicts the result of a study conducted in Korea and Tehran, the capital of Iran, that stated there was no significant relationship between age and user satisfaction in their study [26]. Participants with a monthly income of 88–175 USD group category were more than three times more satisfied than the other group categories with the ambulance service provided. This result is also parallel to the findings in Aboosalehi [8] and Porsuk [3].

Ambulance response time was found to be associated with users’ satisfaction in this study; the narrower the ambulance response time, the higher participant’s satisfaction. However, a great deal of the response time in this study (391 (95.4%)) is below the standard EMS response time, which is < 8 min. These associations between response time and respondents’ views were also reported in García-Alfranca [7], stating that it was the factor that respondents valued most. Reports from Dantas [6–8] and Huabbangyang [6–8] also support this finding. It makes sense to associate this link with speedier responses being more satisfactory to individuals in situations who require immediate assistance.

The other factor that is found to have a significant association with ambulance service satisfaction level in this study is the type of ambulance used, with higher satisfaction in private and NGO ambulance services than in governmental ones. This finding might suggest that the quality of ambulance service given by the private sector and NGOs could be higher than their counterparts, as service user satisfaction level is one of the indicators of service quality. However, the researcher was unable to compare this result with other studies, as previous literature did not study the association between the type of ambulance service used and service satisfaction. This may be due to the organizational, structural, and functional differences in EMS systems between different nations.

In this study, previous ambulance usage was also significantly associated with ambulance service satisfaction, where respondents with previous ambulance usage experience were more than twice as satisfied with the service compared to their counterparts who don’t have previous ambulance usage experience. The findings of this study regarding previous ambulance usage and satisfaction levels correlate with previous studies [6–8]. This literature showed a strong positive relationship between ambulance use experience and satisfaction level. This association between ambulance usage experience and service satisfaction might be explained in terms of familiarity with the service, the more utilization, the higher the satisfaction.

### Limitations of the study

The settings of this study were facility-based, in which clients who used ambulance service to travel to the study settings were asked to respond to an interviewer-administer questionnaire after stabilization of their illness. Therefore, even with multiple data quality control preconditions in place, recall and interviewer bias could be identified as potential limitations to this study. Besides, this study did not include private hospitals, which might have different emergency patient and ambulance flows that affect the generalizability of the data.

## Conclusions

The findings suggest that ambulance personnel performance is a key factor in determining user satisfaction, while treatment on the scene, technical equipment of the ambulance, and call operator sub-scales are areas that require improvement. The study also reveals that age, monthly income, ambulance response time, type of ambulance used, and previous ambulance usage experience are significant factors that influence user satisfaction.

## Data Availability

The data generated and used in this manuscript is available from corresponding author upon reasonable request.
